# Insights into the role of ERp57 in cancer

**DOI:** 10.7150/jca.48707

**Published:** 2021-03-01

**Authors:** Danyang Song, Hao Liu, Jian Wu, Xiaoliang Gao, Jianyu Hao, Daiming Fan

**Affiliations:** 1 Department of Gastroenterology, Beijing Chaoyang Hospital, Capital Medical University, Beijing 100020, China.; 2 State key Laboratory of Cancer Biology, National Clinical Research Center for Digestive Diseases and Xijing Hospital of Digestive Diseases, Air Force Military Medical University, Xi'an 710032, China.

**Keywords:** ERp57/PDIA3, cancer, immune response, immunogenic cell death, unfolded protein response, DNA repair.

## Abstract

Endoplasmic reticulum resident protein 57 (ERp57) has a molecular weight of 57 kDa, belongs to the protein disulfide-isomerase (PDI) family, and is primarily located in the endoplasmic reticulum (ER). ERp57 functions in the quality control of nascent synthesized glycoproteins, participates in major histocompatibility complex (MHC) class I molecule assembly, regulates immune responses, maintains immunogenic cell death (ICD), regulates the unfolded protein response (UPR), functions as a 1,25-dihydroxy vitamin D_3_ (1,25(OH)_2_D_3_) receptor, regulates the NF-κB and STAT3 pathways, and participates in DNA repair processes and cytoskeletal remodeling. Recent studies have reported ERp57 overexpression in various human cancers, and altered expression and aberrant functionality of ERp57 are associated with cancer growth and progression and changes in the chemosensitivity of cancers. ERp57 may become a potential biomarker and therapeutic target to combat cancer development and chemoresistance. Here, we summarize the available knowledge of the role of ERp57 in cancer and the underlying mechanisms.

## Introduction

With cancer incidence and mortality rates increasing rapidly, cancer has become a global problem and is expected to become the leading cause of death worldwide in the 21st century according to the Global Cancer Statistics 2018 report 1. To ease the burden of cancer, current research is focusing largely on molecular preventive trials, which are regarded as the priority study type; biomarkers are key determinants of molecular prevention that allow us to evaluate the natural history of cancer as well as the efficacy and toxicity of an agent 2. Recent research regarding the expression of endoplasmic reticulum resident protein 57 (ERp57) in tumors revealed that ERp57 expression is upregulated in various cancers and participates in cancer initiation, progression and chemosensitivity 3-5. Some evidence suggests that ERp57 can serve as a potential molecular marker and therapeutic target of cancer 6. ERp57 has a molecular weight of 57 kDa and belongs to the protein disulfide-isomerase (PDI) family; it is also referred to as protein disulfide-isomerase A3 (PDIA3), glucose regulatory protein 58 (GRP58) or ER60 protease and is mainly located in the endoplasmic reticulum (ER), with lower amounts in the cytoplasm and nucleus 7, 8. Numerous studies have demonstrated that ERp57 is associated with multiple diseases, such as Alzheimer's disease 9, heart failure 10, thrombus formation 11, 12, neurodegenerative disease and cancer 13. This review summarizes current knowledge relating to the key elements and mechanisms of ERp57 in various tumor functions, such as the regulation of T cell-mediated immune responses, regulation of immunogenic cell death, regulation of the unfolded protein response (UPR), regulation of DNA repair signaling and regulation of membrane-initiated signaling pathways.

## Structure

**Figure 1:**
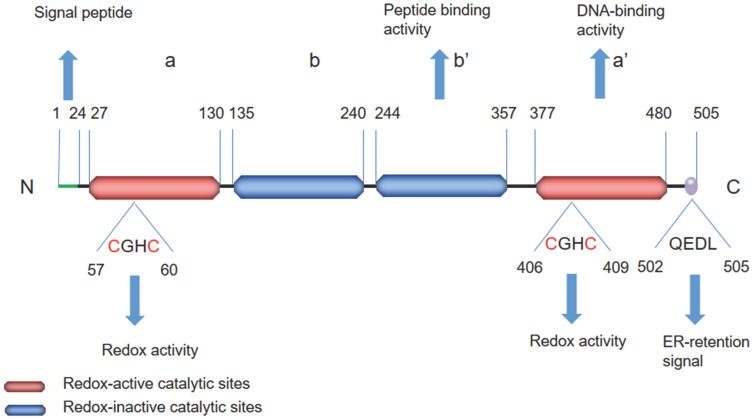
Schematic representation of the structural features of ERp57. ERp57 has four thioredoxin-like site domains—termed a, b, b', and a'—and an acidic C-terminal tail. The C-terminus contains a QEDL sequence that serves as an ER retention signal. The redox-active catalytic sites in the a and a' domains are shown in red, while the redox-inactive catalytic sites in the b and b' domains are shown in blue. The redox-active catalytic sites involve a CGHC sequence. The a' domain has DNA-binding activity, and the b' domain has peptide-binding activity.

**Figure 2:**
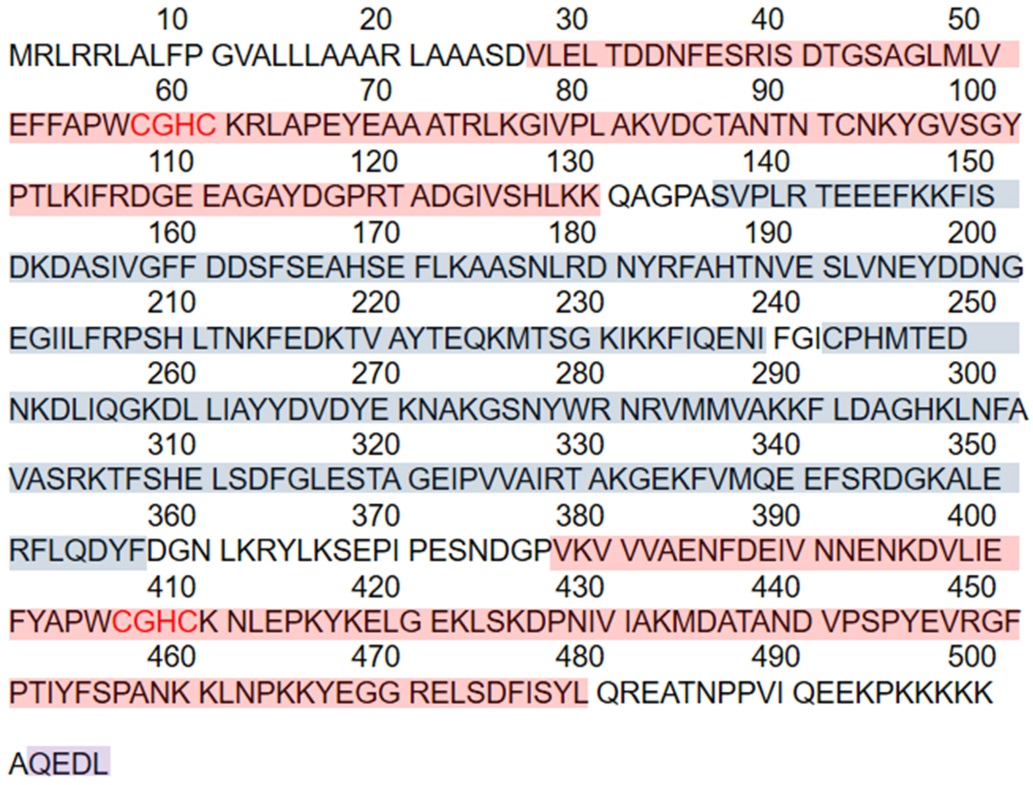
The amino acid sequence of ERp57 and the functional domains. ERp57 has a total of 505 amino acids including four thioredoxin-like site domains, two redox-active catalytic sites, two redox-inactive catalytic sites, and a C-terminal ER retention signal. The redox-active catalytic sites are shown with a red background, while the redox-inactive catalytic sites are shown with a blue background. The C-terminus contains a QEDL sequence shown in purple.

The PDI family includes more than 20 members that catalyze cysteine-based redox reactions and play critical roles in productive protein folding 14, 15. ERp57 is a highly conserved protein consisting of four thioredoxin-like site domains—termed a, b, b', and a'—and an acidic C-terminal tail (Figure 1). Domains a and a' contain the redox active site, whereas domains b and b' are redox inactive. The two thioredoxin-like active domains a and a' each contain a Cys-Gly-His-Cys (CGHC) sequence (Figure 2), and the redox activity of the two domains is provided by the two cysteine residues of the CGHC motif 16, 17. ERp57 exerts reduction, oxidation and disulfide isomerization effects in the ER through thioredoxin-like domains 16, 17. The b' domain contains a ligand-binding site and exhibits high-affinity binding with protein substrates, including both small and large peptide ligands. The b' domain is involved in simple isomerization reactions and directs noncovalent binding to substrates to destabilize the conformation of partially folded species 18. The C-terminus contains a COOH-terminal Gln-Glu-Asp-Leu (QEDL) tetrapeptide that serves as an ER retention signal and acts in cooperation with other amino acid residues 13, 19-21. ERp57 has two general functions: ERp57 binds to double-stranded DNA via recognition of a particular scaffold/matrix-associated region sequence and recruits proteins that localize to the nuclear matrix 22. The DNA-binding properties are associated with the C-terminal region a' domain 23, 24. The affinity of ERp57 for DNA fragments is strongly dependent on the redox state of the two thioredoxin-like sites (i.e., the oxidation of its cysteines) 23, 25. There is a nuclear location sequence in close proximity to its C-terminus; thus, ERp57 can translocate to the nucleus from the cytosol to regulate the transcription of target genes 26, 27. ERp57 exhibits proteolytic activity, degrades other ER proteins and is regulated by acidic phospholipids, including phosphoinositides 19.

## Clinical implications of ERp57 in cancer

**Figure 3:**
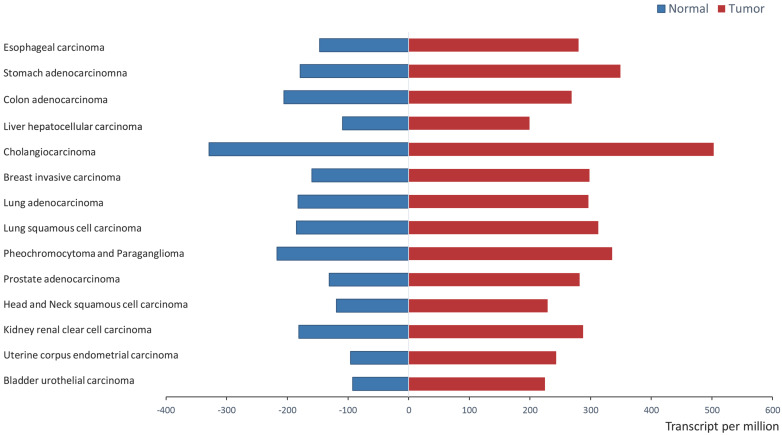
ERp57 expression in normal samples and tumor samples in the TCGA (UALCAN). ERp57 expression is significantly different between tumor samples and tumor samples (*P* < 0.05) according to TCGA genomics data for a wide variety of cancers. The difference in mRNA expression was assessed by Student's t-test.

Recent research from *in vitro* analyses and clinical trials support that ERp57 is overexpressed in a variety of cancers. ERp57 expression is significantly different between tumor tissues and normal tissues (*P* < 0.05) in a wide variety of cancers, including stomach adenocarcinoma, colon adenocarcinoma, liver hepatocellular carcinoma (HCC), breast invasive carcinoma, and prostate adenocarcinoma, as shown in Figure 3. UALCAN analysis (http://ualcan.path.uab.edu/analysis.html) 28 was applied to demonstrate the difference in the mRNA expression of ERp57 between paratumor samples and tumor samples of The Cancer Genome Atlas (TCGA) genomics dataset.

Some studies have demonstrated that ERp57 is a primary cellular target that inhibits cancer cell proliferation 29. ERp57 expression significantly differs among HCC patients, at-risk patients and healthy individuals, thereby potentially serving as a biomarker for the early diagnosis of HCC 6. Evidence suggests that ERp57 is overexpressed in HCC, colorectal cancer 30 and breast cancer 31, 32 and participates in tumorigenesis and the progression of cancer 3, 4. ERp57 expression is associated with the metastatic capacity of cancer cells. ERp57 is overexpressed in 73% of cervical cancers, especially in adenocarcinoma, and high expression indicates poor overall survival and high recurrence-free survival rates in adenocarcinoma patients 33, 34. *In vitro*, knockdown of ERp57 in HeLa cells inhibits cancer invasiveness and metastasis 33, 34. ERp57 levels are also related to the malignant stages of prostate cancer 35. Similarly, high levels of ERp57 in uveal melanoma may indicate that the tumor will metastasize 36. However, ERp57 exerts the opposite effect on gastric cancer progression; its expression is significantly decreased in gastric cancer and metastases, and low levels of ERp57 are correlated with an increased depth of tumor invasion and advanced disease stage 37. The results of retrospective analysis have shown that high expression indicates favorable overall survival in gastric cancer patients 38. ERp57 is also related to gastric cancer treatment. Clinical trials have shown that ERp57 levels in the serum of patients with gastric cancer are decreased significantly after surgical treatment 39. Taken together, these studies demonstrate that ERp57 is associated with tumor initiation and development; however, the implication of ERp57 in cancer is still debated. ERp57 may exert different functions depending on developmental and tissue-specific factors, but it is yet to be understood in detail. Thus, further study to uncover the mechanisms by which ERp57 modulates tumor tumorigenesis and progression is required. Here, we summarize the current research on the biological functions and mechanisms of ERp57 in cancer.

## Biological functions of ERp57 in cancer

### Regulation of T cell-mediated immune responses

Calnexin (CNX) and calreticulin (CRT) predominantly act as lectins that specifically interact with nascently synthesized glycoproteins in the ER, and ERp57 forms complexes with these proteins to accomplish efficient folding and proper formation of intramolecular disulfide bonds in glycoproteins 40, 41. ERp57 binding to substrates is dependent on CNX and CRT and is inhibited by the glucosidase inhibitor castanospermine (CST), an inhibitor of glucosidases I and II 42. ERp57 mediates disulfide bond formation with heavy chains and complexes with CNX 43 to participate in major histocompatibly complex (MHC) class I molecule assembly 44. ERp57 is indispensable for antigen processing and presentation, contributing to the activity of T cell-mediated immune responses. ERp57 is a component of the antigen processing complex that binds to nascently synthesized glycoproteins and facilitates peptide transport from the cytosol into the ER; afterwards, peptides bind to newly synthesized MHC class I molecules to form the MHC class I peptide-loading complex 45. Stable assembly of the MHC class I peptide-loading complex is impaired without ERp57, leading to decreased surface expression of MHC class I peptides 46. Cell surface MHC I expression is essential for the induction of immune responses 47 because MHC class I molecules are involved in antigen presentation and display peptides to circulating CD8+ cytotoxic T cells to enable tumor antigen recognition and induce CD8+ T cell activation, eliciting antitumor immune responses and inducing cancer cell death 48. If MHC class I surface expression is low, cancer cells fail to induce robust immune responses and exhibit weak immunogenicity 49, which is the major mechanism by which tumor cells escape immune attack 50. Moreover, ERp57 expression is associated with CD8+ cytotoxic T lymphocyte dysfunction. The antitumor activity of CD8^+^ T cells is enhanced when ERp57 is knocked out in human CD8^+^ T cells because ERp57 knockout in CD8^+^ T cells regulates the expression of multiple immune regulators and effectors on the cell surface 51. Hence, these reports suggest that ERp57 has a critical role in maintaining antitumor immune responses; thus, it may emerge as a potential cancer therapeutic target for the treatment of tumors for which prior treatment failed to induce strong T cell-mediated immune responses. The mechanism by which ERp57 regulates immune responses and the activity of CD8^+^ T cells need to be explored in detail to expand the utility of ERp57 in cancer immunotherapy strategies.

### Regulation of immunogenic cell death

Immunogenic cell death (ICD), a type of regulated cell death (RCD), induces adaptive immune responses to kill cells directly 52, 53. CRT exposure on the cell surface triggers immune responses and facilitates recognition and removal of apoptotic cells 54, 55. ER stress responses are required for ERp57/CRT exposure on the plasma membrane 56. Knockdown of ERp57 simultaneously inhibits CRT translocation to the surface and exposure, preventing the restoration of tumor cell immunogenicity 57, 58. Many studies have shown that radiation therapy and some chemotherapeutics, in particular anthracycline agents, can induce immune responses and lead to ICD 52, 59. ERp57-deficient cancer cells treated with anthracyclines are unable to elicit anticancer immune responses and become resistant to anthracycline chemotherapy *in vivo* because CRT translocation is inhibited and immunogenic cell death is abolished 58. These studies demonstrate that ERp57 is indispensable for maintaining the immunogenicity of cancer cells and inducing ICD in chemotherapy-treated cells. ERp57 is likely to be a potential indicator of the efficacy of cancer immunotherapy.

### Regulation of the UPR

Due to its disulfide isomerase activity, ERp57 forms a complex with CNX and CRT to specifically modulate the folding of newly synthesized glycoproteins by facilitating the formation of native disulfide bonds 60, 61. In response to intrinsic (oncogene activation) and extrinsic (hypoxia, chemicals or nutrient deficiency) stressors, unfolded and misfolded proteins accumulate in the ER lumen 62. To ease the burden of the ER lumen, the UPR, a finely regulated program, is activated to maintain ER homeostasis and prevent ER stress from inducing death 63. ERp57, as one of the key chaperones of the UPR, is required for this process. Knockdown of ERp57 enhances the ER stress response and facilitates the activation of ATF4/XBP-1 cascades 64. Recent studies have provided evidence that ERp57 maintains the redox state of PDI and that knockdown of ERp57 facilitates PDI oxidation and activates PERK/CHOP cascades to induce p53-dependent apoptosis 65. Moreover, the redox state of ERp57 is very significant. Evidence shows that the action of ERp57 is modulated by ROS because it can induce the oxidative modification of ERp57 and reverse the inhibitory effect on HCC progression 66. In a breast cancer mammosphere model employing SUM159PT cells, mammosphere survival and growth were partially inhibited when ERp57 was knocked down because the effects of the ER protein folding machinery were decreased 67. ER stress plays a critical role in tumor proliferation, progression and resistance, and the UPR alleviates cellular stress and promotes cell survival 68, 69. Recent progress has revealed that ERp57 regulates the UPR, providing valuable information for disrupting the chronic ER stress conditions of cancer. An increasing body of evidence indicates that the UPR may be a target for anticancer therapeutics 70. Studies have reported that ERp57 may be a regulator of the UPR. However, the functional role of ERp57 in the modulation of the UPR in cancer depends on some elements, including the oxidative modification of ERp57, the tumor type, the ER stress conditions and other undetermined factors; thus, more research is required for a deeper understanding.

### Regulation of DNA repair signaling

Recent data suggest that ERp57 plays an important role in the DNA repair process. ERp57, accompanied by high mobility group proteins 1 and 2 (HMGB1 and HMGB2), acts as a component of a complex that detects damaged DNA, inactivates the mismatch repair system and decreases the sensitivity of cells to thiopurine treatment 71, 72. Inhibition of ERp57 significantly abrogates the phosphorylation of H2Aχ and suppresses the induction of the DNA damage response by cytarabine in A549 and UO31 human carcinoma cells 73. Ref-1, a potent activator of p53 74, can regulate p53-mediated base excision repair 75. ERp57 interacts with Ref-1 through its thioredoxin-like domains and cooperates with it to regulate redox-sensitive transcription factor activation, ultimately yielding a protective effect against oxidative insult 76. ERp57, vimentin and the nuclear proteins nucleophosmin (NPM1) and nucleolin (NCL) are major proteins exposed after cell injury; these proteins are necessary for the repair of plasma membrane damage and function as wound-associated proteins 77. Vimentin can protect cells from DNA damage 78, and NPM1 and NCL are DNA damage proteins that directly participate in DNA repair 79, 80. ERp57 has been found to form a complex with vimentin 5, NPM1 and NCL to regulate cell division and cancer development 81; however, the underlying mechanism has not been fully illustrated so far. In addition, ERp57 has roles in chromosome cohesion and segregation; it complexes with β-actin and III β-tubulin (TUBB3) in the nuclear compartment, participating in the regulation of actin conformation states 81 and the attachment of microtubules to chromosomes, which plays a key role in paclitaxel sensitivity 82.

Taken together, the evidence indicates that ERp57 has a protective effect on the survival of tumor cells due to its contribution to the DNA repair process, which regulates the sensitivity of the tumor to DNA-modifying chemotherapy. Thus, it may become a biomarker to predict therapeutic failure and success. However, further studies are required to confirm these findings and identify potential antitumor targets.

### Regulation of membrane-initiated signaling pathways

1,25(OH)_2_D_3_ is the active form of vitamin D, and its analogs exert inhibitory effects on the proliferation and progression of multiple cancers, including melanoma 83, prostate cancer, and leukemia 83, 84. ERp57 acts as a 1,25D_3-_membrane-associated, rapid response to steroid binding (1,25D_3_-MARRS) receptor, mainly located on the plasma membrane and Golgi/ER 85-87. It binds to secosteroid 1,25(OH)_2_D_3_ to form a ligand-receptor complex to mediate several rapid signal transduction cascades; for example, it regulates STAT3 88 and the NF-κB pathway 89, promotes the formation of complexes involving ERp57 and other proteins, and translocates to the nucleus to regulate gene expression 27. ERp57 complexes with NF-κB to activate downstream cascades to regulate leukemia cell differentiation 89. ERp57 regulates the transcription and translation of STAT3 through binding to STAT3 DNA and protein 90 and regulates the level of STAT3 phosphorylation to modulate the transcriptional activity of STAT3, which affects gene expression 91, 92. The ERp57/STAT3 complex translocates to the nucleus to induce MCOLN3 expression and enhance Ca^2+^ release from lysosomes to activate autolysosomal degradation 93. *In vitro* studies have revealed that knockdown of ERp57 inhibits HCC cell proliferation 92 and enhances the paclitaxel sensitivity of paclitaxel‑resistant SKOV3/tax cells by blocking the STAT3 pathway 5. Furthermore, a study reported that knockdown of ERp57 prevents EGFR-mediated cancer proliferation by inhibiting the internalization and phosphorylation of the EGF receptor 94. EGFR amplification or mutation facilitates tumor proliferation, invasion, migration and apoptosis inhibition by regulating multiple downstream cascades, and EGFR inhibitors provide an effective therapeutic target for EGFR-mediated cancer 95. Thus, analysis of the inhibitory function of ERp57 on EGFR-mediated cancer could open a new avenue to further explore optimized treatment options.

### Regulation of other signal transduction pathways

Mitomycin C (MMC) exerts anticancer effects by inducing DNA cross-linking in cancer cells 96. ERp57 functions as a reductase, catalyzes the metabolic reduction of MMC and induces DNA cross-linking via its two thioredoxin-like domains 97, leading to increased MMC cytotoxicity 96. MMC cytotoxicity induced by DNA cross-linking and breakage is also associated with numerous side effects, such as lack of appetite, weight loss, and renal toxicity, which are alleviated after inhibition of ERp57 98.

Affymetrix microarray results suggest that cytoskeletal remodeling and the Wnt signaling pathway are the two of the top pathways disrupted by ERp57 silencing in HeLa cells 99. The present data demonstrate that β-catenin participates in the mechanisms of cell adhesion, cytoskeleton remodeling and the canonical Wnt signaling pathway 100. Stable knockdown of ERp57 induces β-catenin accumulation and inhibits the migration of HeLa cells 99, and resveratrol can increase the interaction between ERp57 and β-catenin 101. In addition, ERp57 can regulate the proliferation, invasion and migration of cancer via the MAPK signaling pathway. Downregulation of ERp57 suppresses JNK, ERK and p38 phosphorylation 102, ERp57 expression requires ERK and p38, and inhibition of the ERK/p38 MARK pathway suppresses ERp57 expression 98. Little is known regarding the regulatory role of ERp57 in these pathways, and more research is needed to confirm these findings.

## Conclusions

**Figure 4:**
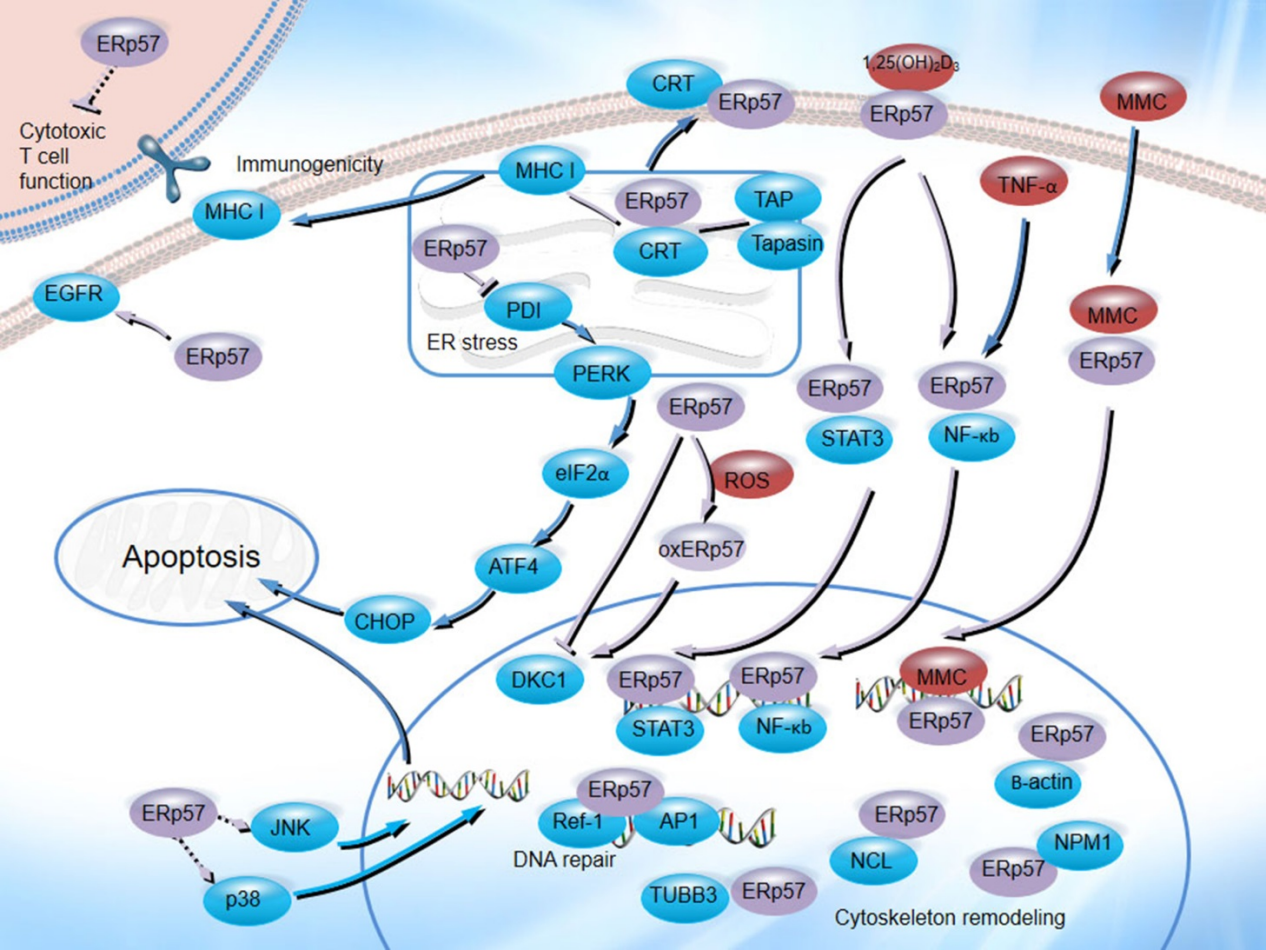
The biological functions of ERp57 in cancer. ERp57 regulates diverse signal transduction pathways: ERp57 is associated with CRT and CNX, participates in MHC class I molecule assembly, inhibits T cell-mediated immune responses, regulates the UPR, functions as a receptor for 1,25(OH)_2_D_3_, binds to STAT3 and NF-κB, enhances MMC-induced DNA cross-linking, and participates in DNA repair processes and cytoskeletal remodeling.

ERp57 plays an important role in the natural history of cancer and the therapeutic process. As an ER chaperone, ERp57 is associated with CRT and CNX, participates in MHC class I molecule assembly 103, 104, regulates tumor cell immunogenicity 105, facilitates new glycoprotein synthesis and enhances oxidative refolding of denatured cells 106, functions as a 1,25D_3_-MARRS receptor 107, regulates gene transcription, binds to STAT3 and NF-κB, enhances MMC-induced DNA cross-linking and is involved in DNA repair processes and cytoskeletal remodeling (Figure 4). Numerous studies have demonstrated that ERp57 plays a significant role in tumor development and chemoresistance in a wide variety of cancers. These identified crucial roles and their regulatory pathways may provide a framework for the comprehensive understanding of ERp57 in cancer, which might indicate its potential as a potential therapeutic target for cancer treatment.

## Abbreviations

ERp57: Endoplasmic reticulum resident protein 57
PDIA3: protein disulfide-isomerase A3
PDI: protein disulfide-isomerase
ER: endoplasmic reticulum
MHC: major histocompatibility complex
ICD: immunogenic cell death
UPR: unfolded protein response
1,25(OH)_2_D_3_: 1,25-dihydroxy vitamin D_3_
GRP58: glucose regulatory protein 58
CGHC: Cys-Gly-His-Cys
QEDL: Gln-Glu-Asp-Leu
HCC: hepatocellular carcinoma
TCGA: The Cancer Genome Atlas
CNX: calnexin
CRT: calreticulin
CST: castanospermine
RCD: regulated cell death
HMGB1: high mobility group proteins 1
HMGB2: high mobility group proteins 2
NPMI: nucleophosmin
NCL: nucleolin
TUBB3: III β-tubulin
1,25D_3_-MARRS: 1,25D_3_-membrane-associated, rapid response to steroid binding
MMC: Mitomycin C
